# Adding Tat‐Beclin1 to an endurance training program promotes additive improvements in physical function in aged male mice

**DOI:** 10.14814/phy2.71128

**Published:** 2026-10-09

**Authors:** Thuan Thien Tchen, Shakibur Rahman, Thaysa Ghiarone, Lynn A. Spruce, Hossein Fazelinia, Elizabeth M. Brown, Charalampos Papachristou, Sue C. Bodine, Vitor A. Lira, Kleiton A. S. Silva

**Affiliations:** ^1^ Department of Biomedical Sciences Cooper Medical School of Rowan University Camden New Jersey USA; ^2^ School of Translational Biomedical Engineering and Sciences (TBES), Rowan University Stratford New Jersey USA; ^3^ Proteomics Core Facility Children's Hospital of Philadelphia Philadelphia Pennsylvania USA; ^4^ Department of Biomedical and Health Informatics Children's Hospital of Philadelphia Philadelphia Pennsylvania USA; ^5^ Department of Mathematics Rowan University Glassboro New Jersey USA; ^6^ Aging and Metabolism Research Program Oklahoma Medical Research Foundation Oklahoma City Oklahoma USA; ^7^ Department of Health, Sport, and Human Physiology, Fraternal Order of Eagles Diabetes Research Center, Pappajohn Biomedical Institute University of Iowa Iowa City Iowa USA

**Keywords:** autophagy, exercise, physical function, proteomics, Tat‐Beclin1

## Abstract

Autophagy is a hallmark of aging, but it has not been exclusively targeted to attenuate aging‐related physical dysfunction. Here, we combined Tat‐Beclin1 and endurance training to determine its effect on physical function domains in aged male mice. Mice were randomly divided into Control (*N* = 5), Tat‐Beclin1 (TB, 15 mg/kg, 2×/week, *N* = 9), Exercise (Exe, 70%‐maximal‐running‐speed, 3×/week, *N* = 9), and TB+Exe (*N* = 8) for 1 month in 23‐month‐old male C57BL/6J mice. Animals were assessed for grip strength (GS), endurance capacity (EC) on a treadmill, and balance and coordination on a rotarod. Gastrocnemius/plantaris (G/P) and tibialis anterior muscles were harvested for western blotting, myofiber typing, and proteomic profiling (G/P only). Although no significant interaction was observed, the TB+Exe group showed improvements in GS, EC, and balance and coordination performance, an outcome comparable with additive effects of both therapies. Limited changes were observed in myofiber typing and autophagy markers among groups. A proteomic analysis revealed that TB upregulated biological processes involved in muscle contraction, whereas TB+Exe surprisingly upregulated acute inflammatory responses, including proteins such as haptoglobin and orosomucoid‐1. Altogether, combining TB and endurance training elicits additive effects on physical functions of aged male mice and may represent a new approach to attenuate aging‐related physical dysfunction.

## 1 INTRODUCTION

Aging leads to a progressive decline in physical function, negatively impacting the quality of life of affected individuals (Cruz‐Jentoft et al., 2019; Izquierdo et al., 2021; Izquierdo & Fiatarone Singh, 2023; Murray et al., 2026; Tieland et al., 2018). Given that the world population is aging at an alarming pace (Dzau & Jenkins, 2019; United Nations, 2024), developing therapies improving overall physical function is essential for extending healthspan (Elliehausen et al., 2023). Regular exercise is an effective approach for slowing aging‐related deterioration in physical function, but limitations, including physical debilitation and age‐associated diseases, prevent many older adults from adhering to an exercise program (Carrick‐Ranson et al., 2022). Intriguingly, even among long‐term exercise practitioners, the negative effects of aging may limit the benefits of exercise (Carrick‐Ranson et al., 2022; Cruz‐Jentoft et al., 2019). Several factors contribute to the development and progression of functional decline, including insulin resistance, mitochondrial dysfunction, chronic low‐grade inflammation, impaired proteostasis and macroautophagy (hereafter referred to as autophagy), and cardiovascular dysfunction (Carnio et al., 2014; Ko et al., 2016; Lopez‐Otin et al., 2023). In an attempt to identify a drug that attenuates aging‐induced physical dysfunction, many pharmacological agents, including metformin, rapamycin, vitamin D, testosterone, SS‐31, and MitoQ (De Spiegeleer et al., 2018; Elliehausen et al., 2023; Ham et al., 2022; Murray et al., 2026; Petrocelli et al., 2023), have shown promising results in reducing aging‐related physical dysfunction. Conversely, when testing some of these agents in combination with regular exercise, available data suggest that exercise adaptations are limited or blunted by the combinatory approach (Bodine et al., 2001; Bruss et al., 2025; Konopka et al., 2019; Walton et al., 2019). Therefore, it is imperative to expand our understanding of the mechanisms that modulate aging‐related physical dysfunction and to identify effective therapies that may enhance the benefits of exercise.

Autophagy is the primary cellular mechanism for degrading long‐lived proteins and organelles and is essential for opposing physical dysfunction (Aman et al., 2021; Masiero et al., 2009). It has been postulated that autophagic activity declines with aging (Lopez‐Otin et al., 2023), which can be rescued by aerobic exercise, benefiting aged muscles and hearts (Kim et al., 2013; Liang et al., 2021; Pinto et al., 2021; Zeng et al., 2020). This makes autophagy a suitable target to counteract age‐related declines in physical function. Although pharmacological agents that attenuate physical dysfunction may modulate autophagy, they do so by targeting molecules that are not constitutively embedded in the autophagy machinery (i.e., autophagy‐related proteins). Beclin1 is a fundamental autophagy‐related protein that coordinates autophagosome formation and fusion with lysosomes through its association with the class III phosphatidylinositol 3‐kinase (PtdIns3K) complex (Levine et al., 2015). The role of Beclin1 in maintaining physical function has been demonstrated in a young Beclin1 haploinsufficient mouse model, in which Beclin1 deficiency blunted exercise‐induced increases in capillary density and endurance capacity (Lira et al., 2013). Additionally, in a transgenic mouse model with a BECN1^F121A^ knock‐in mutation that activates autophagy by disrupting the Beclin1/BCL2 interaction, myofiber cross‐sectional area was preserved in 20‐month‐old mice (Sebti et al., 2022). These data demonstrate that genetically targeting Beclin1 is sufficient to alter exercise adaptation and improve aging‐related muscle dysfunction. Nevertheless, only one study has pharmacologically targeted Beclin1 (i.e., Tat‐Beclin1) in a mouse model with sustained mTOR activation, showing that activation of autophagy improves mitochondrial function and prevents neuromuscular junction dysfunction (Baraldo et al., 2020). Tat‐Beclin1 is a small permeable peptide derived from the Beclin1 protein (Shoji‐Kawata et al., 2013). Mechanistically, Tat‐Beclin1 binds to the Golgi‐associated plant pathogenesis‐related protein 1 (GAPR‐1), a negative regulator of autophagy, releasing Beclin1 back into the PtdIns3K complex and triggering autophagy (Shoji‐Kawata et al., 2013; Zhao et al., 2021). Yet whether Tat‐Beclin1 serves as a pharmacological agent counteracting age‐related physical dysfunction remains elusive.

In this study, we hypothesized that combining Tat‐Beclin1 and endurance training in an alternating‐day regimen would yield superior and synergistic benefits in physical function domains (i.e., grip strength, endurance capacity, and balance and coordination) compared with single‐mode treatments and would identify key molecules associated with improvements in physical function. We find that Tat‐Beclin1 and exercise independently improved grip strength, endurance capacity, and balance and coordination. Although no significant interaction was observed, combining Tat‐Beclin1 and endurance training improved gains in grip strength, endurance capacity, and balance and coordination, an outcome comparable with additive effects of both therapies. Additionally, our proteomics analysis showed that there was an overall overlap of fatty acid metabolism upregulation from each therapy compared to control animals. In addition, the combinatorial strategy increased mitochondrial bioenergetic processes and unexpectedly upregulated proteins involved in the acute inflammatory response, revealing a complex and novel regulatory mechanism that attenuates skeletal muscle aging. Taken together, our findings demonstrate that Tat‐Beclin1 is a promising candidate for attenuating aging‐related physical dysfunction and add to the beneficial effects of exercise when combined with endurance training.

## 2 MATERIALS AND METHODS

### 2.1 Animals

Male C57BL/6J mice were purchased from the Jackson Laboratory (Bar Harbor, ME) at 22 months of age, which is roughly equivalent to 69 years old in human age. After 2 weeks, at 22.5 months of age, animals were used for experimental purposes (i.e., physical function assessments). Animals were randomly housed in groups of 3–5 mice per cage. Housing conditions included an enriched cage environment, a standard chow diet (5LG4—LabDiet® JL Rat and Mouse /Irr 6F, Richmond, IN), and water ad libitum, with room temperature controlled at 21°C–22°C, and light and dark cycles of 12 h each. Mice were randomly divided into Control (*N* = 5), Tat‐Beclin1 (TB, *N* = 9), exercise (Exe, *N* = 9), and TB+Exe, *N* = 8 (Figure 1). Forty‐eight hours after the last Tat‐Beclin1 injection (TB group) or the last exercise session (Exe and TB+Exe groups), all animals were euthanized between 1300 and 1700 h during the light cycle by CO_2_ overdose followed by cervical dislocation. All animal protocols were performed under the Principles for the Utilization and Care of Vertebrate Animals Used in Testing, Research, and Training and approved by the Rowan University Animal Care and Use Committee (Protocol No. 2022‐1316).

**FIGURE 1:**
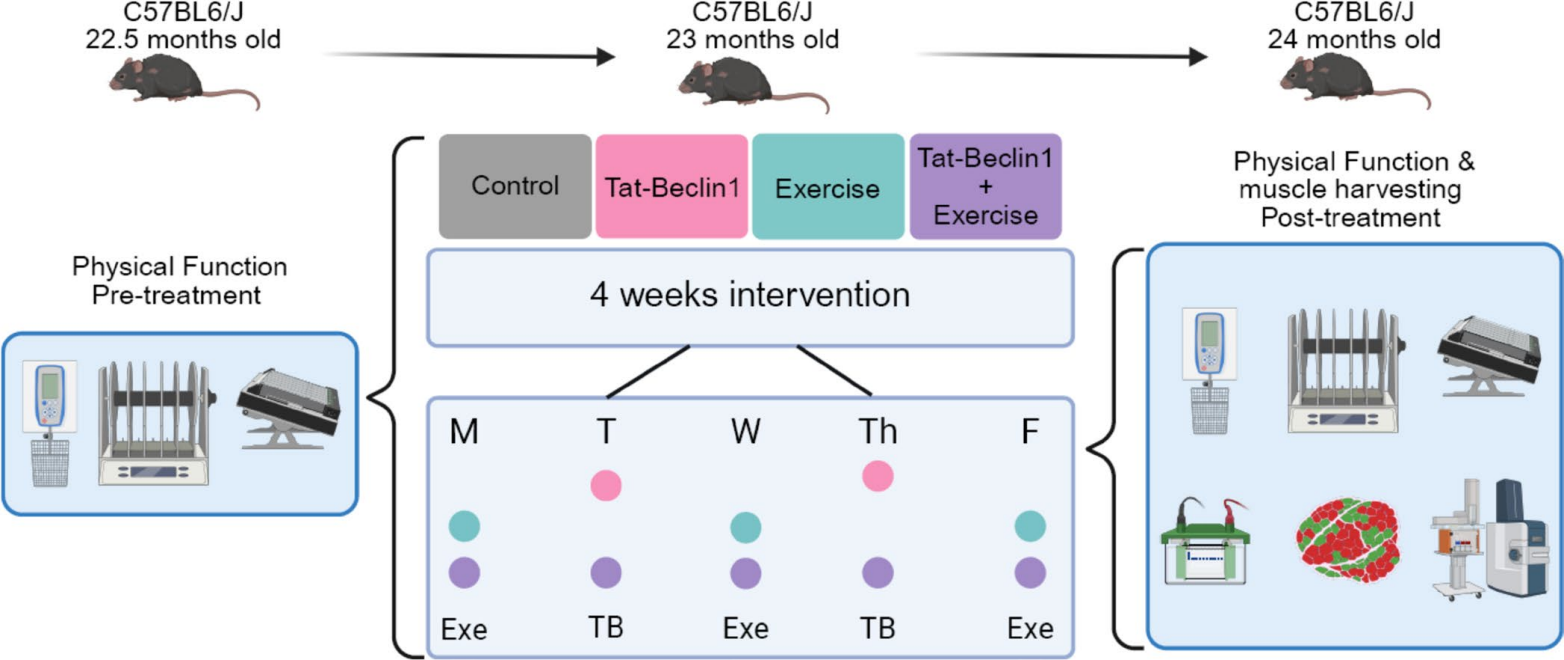
Experimental design. Mice underwent physical function testing before the intervention, including a familiarization period followed by baseline testing. Twenty‐three‐month‐old male C57BL6/J mice were assigned to Control, Tat‐Beclin1 (TB), Exercise (Exe), and TB+Exe groups and completed a 4‐week intervention in an alternating‐day regimen. Exercise was performed three times per week (Monday, Wednesday, and Friday), and Tat‐Beclin1 was administered two times per week (Tuesday and Thursday). After the intervention, mice underwent post‐treatment physical function testing, and muscles were harvested for morphological, protein‐expression, and proteomic analyses.

### 2.2 Physical function

We conducted physical function measurements at the pre‐ and post‐treatment periods. All tests were conducted between 1300 and 1700 h during the light cycle. Animals were familiarized with a grip strength meter (Cat. No. 76‐1068), a motorized treadmill (Cat. No. LE8710MTSAPC), and a rotarod (Cat. No. LE8205), all purchased from DSI/Panlab/Harvard Apparatus. Mice were familiarized with the grip strength meter apparatus 24 h prior to the actual test. Each mouse was subjected to five trials, during which they were positioned with the four paws on the grid and gently pulled back. On the day of the test, mice were positioned with all four paws on the grid, and a designated investigator lightly but securely held each mouse by the base of the tail. After confirming that the animal had grasped the grid, the investigator gently pulled the animal back. The grip strength of the mouse's four paws was recorded in Newtons (N). The grip strength test was performed using four paws due to generalized effects of aging on virtually all muscles (Cruz‐Jentoft & Sayer, 2019), best assessing the effects of aging on multiple muscles. We conducted five trials per animal, allowing 1 min of rest between trials (Bonetto et al., 2015). For the rotarod test, mice were familiarized to the rotarod 24 h before testing. The training test consisted of five trials, in which each mouse was placed on a stationary rod for 1 min. Next, animals were placed on the rod at 5 rpm for 1 min per trial, up to 4 trials in total. On the day of the test, animals performed three trials, each consisting of an accelerating rotarod set at a gradual speed (i.e., 4–40 rpm) for 5 min (Justice et al., 2014). The latency to fall (in seconds) was recorded. After completing the grip strength and rotarod assessments, animals were familiarized with a motorized treadmill as follows: Day 1: stationary treadmill belt at 10% slope for 10 min; Day 2: 5 m/min for 5 min at 10% slope; Day 3: 5 m/min for 5 min at 15% slope; Day 4: 5 m/min for 5 min at 20% slope; Day 5: 5 m/min for 5 min at 25% slope; Day 6: resting; and Day 7: incremental test, which served as an evaluation of endurance capacity and for training prescription. The incremental test consisted of a warm‐up period at 5 m/min for 3 min at a 25% slope; the speed was then increased by 5 m/min every 1 min until exhaustion (Cho et al., 2021). Failure to maintain treadmill speed was characterized by contact with the tail or hindlimbs on the back of the acrylic lane, or by standing or sitting on the grid, which elicited a grid shock (0.2 V) for approximately 5 s. Endurance capacity was reported as time to exhaustion in seconds (Murray et al., 2026).

### 2.3 Endurance training

At 23 months of age (Figure 1), animals in the Exe and TB+Exe groups underwent 1 month of endurance training. Although animals from the Control and TB groups did not participate in exercises, they were brought to the exercise room and positioned near the treadmill to account for stress associated with treadmill operation. Exercise sessions were held between 1300 and 1700 h during the light cycle on Mondays, Wednesdays, and Fridays, for a total of 12 sessions. Each session included a warm‐up period of 5 m/min for 5 min, a training period of 33 min (during the first and second weeks) and 43 min (during the third and fourth weeks) at 70% of the maximum running speed, and a cool‐down period of 5 m/min for 2 min. The treadmill slope was maintained at 5% in the first week and increased to 10% for the remainder of the protocol.

### 2.4 Tat‐Beclin1 administration

Tat‐Beclin1 was obtained from Novus Biologicals (Cat No. NBP2‐49888). Tat‐Beclin1 was reconstituted in sterile 1× phosphate‐buffered saline (PBS). Mice in the TB and TB+Exe groups received intraperitoneal injections of 15 mg/kg of Tat‐Beclin1 between 1300 and 1700 h during the light cycle on Tuesdays and Thursdays, totaling 8 injections (Figure 1). Control and Exe groups were given PBS as a control for drug injection and to account for stress from the animal handling procedure. The chosen Tat‐Beclin1 dose was supported by studies highlighting its efficacy as an autophagic inducer (Schirone et al., 2026; Shoji‐Kawata et al., 2013; Soria et al., 2021).

### 2.5 Protein extraction, western blotting, and antibodies

Forty‐eight hours after the last TB injection or the last exercise session, gastrocnemius/plantaris (G/P) and tibialis anterior (TA) muscles were harvested, weighed, flash‐frozen in liquid nitrogen, and stored at –80°C. Cryo‐pulverized G/P and TA muscles were homogenized in a mix of ice‐cold RIPA buffer (Thermo Fisher Scientific, Waltham, MA; Cat. No. 89901) and protease and phosphatase inhibitor cocktail (Thermo Fisher Scientific, Waltham, MA; Cat. No. 78441). Homogenates were centrifuged at 15,000 g for 15 min at 4°C. We used a BCA Protein Assay Kit (Thermo Fisher Scientific, Waltham, MA; Cat. No. 23227) to measure total protein concentration. Ten or fifteen micrograms of total protein were loaded in an SDS‐PAGE gel and transferred to a 0.22 μm or 0.45 μm PVDF membrane. Membranes were blocked with 5% skim milk in 1× PBS + 0.1% Tween‐20 for 1 h at RT, followed by overnight incubation with primary antibodies in a similar solution. Primary antibodies used in this study include microtubule‐associated protein 1 light chain 3B (LC3B, 1:1000, No. ab192890), Beclin1 (1:2000, No. ab207612) from Abcam (Cambridge, UK), Sequestosome1/p62 (SQSTM1/p62, 1:1000, No. 23214) from Cell Signaling Technology, and Orosomucoid‐1 (ORM1, 1:1000, No. MA5‐24279) and Haptoglobin (HP, 1:1000, No. MA5‐32919) from Invitrogen. Since the liver is the primary tissue synthesizing ORM1 and HP (Fournier et al., 2000; Kushner et al., 1981), we conducted a pilot experiment in livers harvested from animals in this study (Figure S1a,b). Membranes were washed 3x for 10 min each in 1× PBS + 0.1% Tween‐20, then incubated with appropriate secondary antibodies (Goat anti Rabbit IgG, 1:3000, No. 7074S; Horse anti Mouse IgG, 1:3000, No. 7076S; Goat anti Rat IgG, 1:3000, No. 7077S from Cell Signaling) diluted in 1× PBS + 0.1% Tween‐20. We then developed the membranes by immersing them in ECL (ThermoFisher Scientific, No. 32106). Since aging can alter the protein expression of traditional housekeeping proteins, including GAPDH and α‐actin (Vigelso et al., 2015), we used Coomassie Brilliant Blue G 250 (ThermoFisher Scientific, No. 20278) as a loading control and normalization method (Welinder & Ekblad, 2011).

### 2.6 Immunofluorescence

G/P and TA muscles were harvested, placed onto a cork, and submerged in liquid nitrogen‐cooled isopentane for ~30 s and stored at −80°C. When ready, we collected two muscle sections (10 μm) per slide for each animal and analyzed skeletal muscle (i.e., gastrocnemius/plantaris and tibialis anterior), with sections within 100 μm of each other. Sections were collected using Epredia™ Shandon™ ColorFrost™ Plus Slides (ThermoFisher Scientific, No. 9991001) and stored at −80°C until ready for staining. Next, sections were air‐dried for 20 min at room temperature, washed in PBS for 5 min, and blocked in 10% goat serum (No. G9023) from Sigma‐Aldrich, 0.05% Triton X‐100, and PBS for 1 h at RT. After washing sections with PBS for 5 min, 3× each, samples were incubated for 2 h at RT with SC‐71 (mouse IgG1, 1:400) to detect myosin heavy chain MyHC 2a, and BF‐F3 (mouse IgM, 1:50) to detect MyHC 2b, purchased from the Developmental Studies Hybridoma Bank, and Laminin (Ab11575, rabbit IgG, 1:100) from Abcam. Following primary antibody incubation, muscle sections were washed with PBS (5 min, 3×) and incubated with secondary antibody as follows: Alexa Fluor 488 (MIgG1, 1:200) (No. A21121), Alexa Fluor 555 (MIgM, 1:200) (No. A21426), and Alexa Fluor 350 (RbIgG (H+L), 1:200) (No. A11046) from Invitrogen for 1 h at RT. ProLong™ Gold Antifade Mountant (ThermoFisher Scientific, No. P36930) was applied to the sections, and a coverslip was applied. Slides were immediately imaged or stored at 4°C. Multichannel images of the G/P and TA muscle sections were captured using a Leica DM4000 B LED microscope equipped with a K3M camera. High‐resolution images of the entire muscle section were assembled using the Grid/Collection stitching plugin in Fiji in sequential‐image mode. Next, multichannel fluorescent images were segmented using Cellpose Sam, and the resulting masks were imported into the LabeltoROIs plugin in Fiji, alongside the original images, to accurately quantify myofiber features. The data generated by this process were subsequently analyzed using a custom Python script for detailed processing.

### 2.7 Proteomics

Cryopulverized G/P muscle tissue was lysed, solubilized, and digested on an S‐Trap Micro (Protifi). The resulting peptides were de‐salted using an Oasis HLB μElution plate (Waters), dried via vacuum centrifugation, and reconstituted in 0.1% TFA containing iRT peptides (Biognosys Schlieren, Switzerland). Peptides were analyzed using a QExactive HF mass spectrometer (Thermo Fisher Scientific, San Jose, CA) coupled with an Ultimate 3000 nano UPLC system and an EasySpray source, employing data‐independent acquisition (DIA). The raw mass spectrometry data were searched using direct‐DIA mode in Spectronaut. The MS2 intensity values for proteins generated by Spectronaut were used for bioinformatics analysis.

### 2.8 Statistical analyses

Data are presented as means ± standard deviation (SD). The Shapiro–Wilk normality test was applied to assess whether the data were normally distributed. The data were not normally distributed; thus, we used nonparametric tests throughout the manuscript. A paired‐sample *t*‐test was applied for pre‐ and post‐assessments. Two‐way ANOVA was performed to compare statistical differences among groups, and results were reported as main effect and interaction. When interaction reached statistical significance (α < 0.05), a Tukey's multiple comparisons test was applied to detect differences among groups. Proteomics intensities were filtered to remove proteins with excessive missingness and normalized using sample‐wise median centering to reduce technical variation. Differential protein abundance between groups was evaluated using linear modeling with empirical Bayes variance moderation as implemented in the limma framework, a widely used method for high‐dimensional omics data that improves variance estimation and statistical power. Multiple testing correction was performed using the Benjamini–Hochberg (BH) procedure, and significantly differentially expressed proteins were subsequently examined using volcano plots and hierarchical clustering heatmaps. To assess pathway‐level enrichment patterns, we performed Gene Set Enrichment Analysis (GSEA) using curated KEGG pathway gene sets. Briefly, we determined enrichment of KEGG pathways and Gene Ontology (GO) terms related to biological processes (BP) by ranking detected proteins based on log fold‐change. The enriched pathways were then plotted as GSEA – GROUP X versus GROUP Y for GO:BP. The graphics show 10 upregulated processes with the highest normalized enrichment score (NES), and the 10 downregulated processes with the highest negative NES. We also showed two levels of false discovery rate (FDR): ≤0.05, >0.05, and ≤0.1.

## 3 RESULTS

### 3.1 Impact of Tat‐Beclin1 and endurance training on body and muscle weight

We assessed the mouse's body weight pre‐ and post‐treatment. Each therapy group caused a decrease in body weight at post‐treatment (TB, *p* = 0.0014, −12.11%; Exe, *p* = 0.018, −8.69%; and TB+Exe, *p* = 0.015, −5.29%) compared to pre‐treatment (Figure 2a). While a significant interaction (*p* = 0.0102) was found in the net body weight, a Tukey's multiple comparisons test showed no difference among groups (Figure 2b). In addition, we found no significant differences in main effects or interactions in the muscle masses of G/P, TA, extensor digitorum longus, and soleus (Figure 2c–f; *p* > 0.05).

**FIGURE 2:**
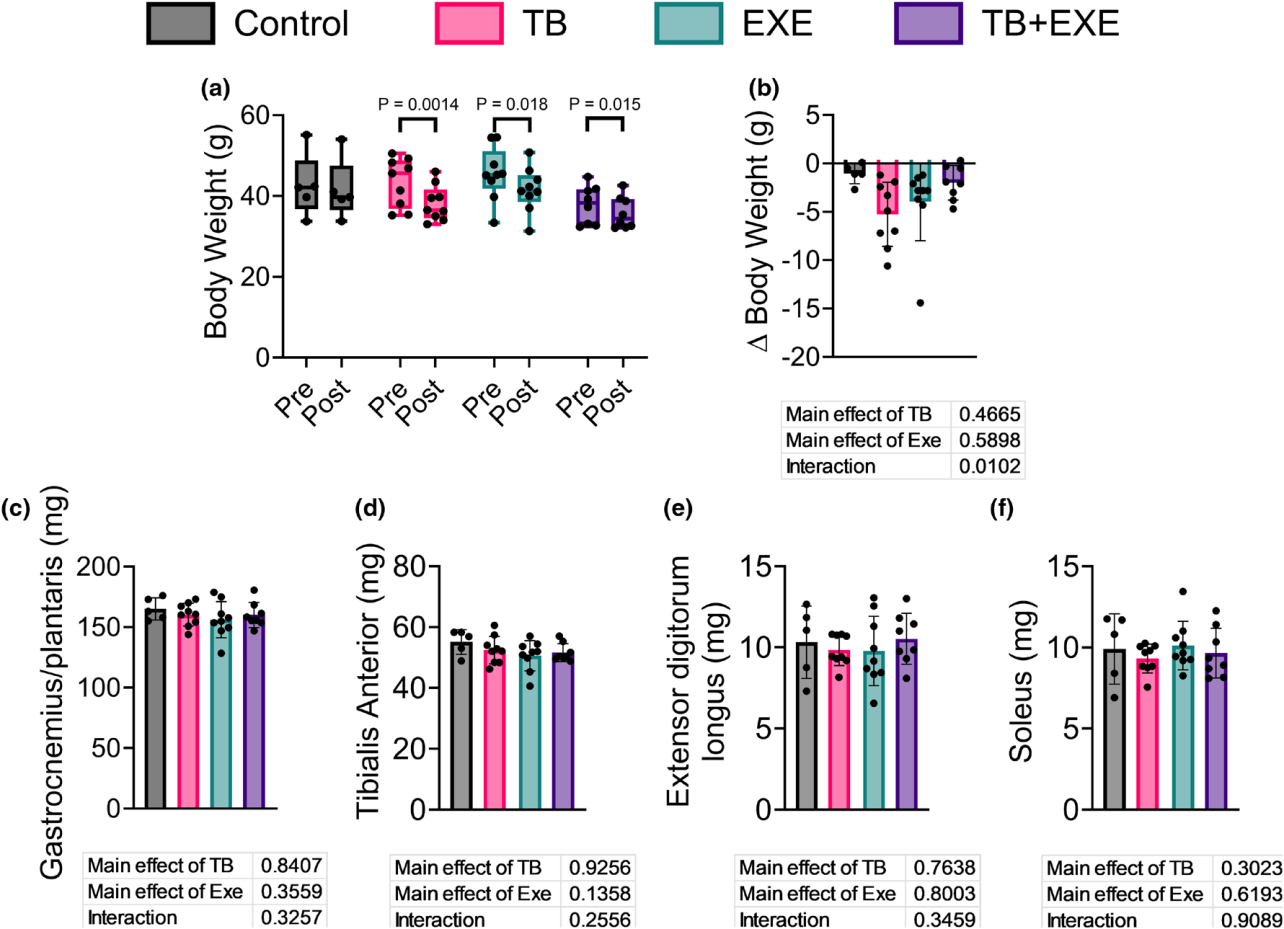
Body weight and hindlimb muscle masses following Tat‐Beclin1 and endurance training. (a) Body weight (g) was measured before and after the intervention in Control, Tat‐Beclin1 (TB), Exercise (Exe), and TB+Exe groups. (b) Change in body weight (Δ body weight, g). (c–d) Wet muscle masses (mg) for gastrocnemius/plantaris (c), tibialis anterior (d), extensor digitorum longus (e), and soleus (f). Data are presented as mean ± standard deviation with individual data points shown. For body weight measured pre‐ and post‐intervention, statistical analysis was performed using a paired sample t‐test; data were considered statistically significant with an α < 0.05. Δ body weight and muscle masses were analyzed by two‐way ANOVA; data were considered significant for main effects or interaction with an α < 0.05. A Tukey's multiple comparison analysis was conducted when interaction was statistically significant. 23‐ to 24‐month‐old male C57BL6/J mice: Control, *N* = 5; TB, *N* = 9; Exe, *N* = 9; TB + Exe, *N* = 8.

### 3.2 Tat‐Beclin1 and endurance training effects on aging‐related physical function domains

To determine whether TB, or its addition to a short‐term endurance training regimen, affects physical function in aged male mice, we measured grip strength, endurance capacity on a treadmill (time to exhaustion), and balance and coordination on a rotarod. Evaluating the effects of the one‐month intervention on grip strength, our data revealed that TB and TB+Exe groups increased grip strength post‐treatment compared with pre‐treatment (Figure 3a; +20.43%, *p* = 0.048; +41.74%, *p* < 0.0001, respectively), whereas no difference was observed in the control (Figure 3a; −16.51%, *p* > 0.05) or Exe (Figure 3a; +1.37%, *p* > 0.05) groups. Regarding the net outcome, we found a main effect of TB (*p* = 0.0003) and Exe (*p* = 0.0224). The combination of the two therapies did not lead to a significant interaction; it caused a higher gain in the TB + Exe group (Figure 3b: 0.401 N) compared to TB (0.185 N) and Exe (0.015 N), an outcome comparable with additive effects of both therapies. Next, we conducted an incremental test to assess endurance capacity. While the control group showed a trend of decreasing endurance capacity (Figure 3c, *p* = 0.053, −21.17%), all treated groups showed improvements in the test (Figure 3c, TB, *p* = 0.016, +37.41%; Exe, *p* < 0.0001, +53.87%; and TB+Exe, *p* < 0.0001, +66.67%). The net gain shown in Figure 3d revealed main effects of TB (*p* = 0.0033) and Exe (*p* < 0.0001), and similar to grip strength, no significant interaction (*p* = 0.0787) was observed. The gain in endurance capacity in the TB + Exe group is an outcome comparable with additive effects of both therapies (Figure 3d, 506.85 s compared to TB 234.13 s and Exe 336.20 s). Because aging increases the risk of falls and fractures, we sought to determine whether our short‐term therapeutic approach improves mice's balance and coordination by conducting a rotarod assay. Compared with the pre‐treatment period, the control group showed a significant decrease (Figure 3e, *p* = 0.011, −22.41%), and the TB+Exe group showed an increase of +60.28% (*p* = 0.008), while the TB and Exe groups showed no difference. Additionally, significant main effects of TB (*p* = 0.0397) and Exe (*p* = 0.0379) were observed. Although no significant interaction (*p* = 0.5473) was observed, the gains in balance and coordination in the TB+Exe group indicate an outcome comparable to the additive effects of both therapies in this physical function domain.

**FIGURE 3:**
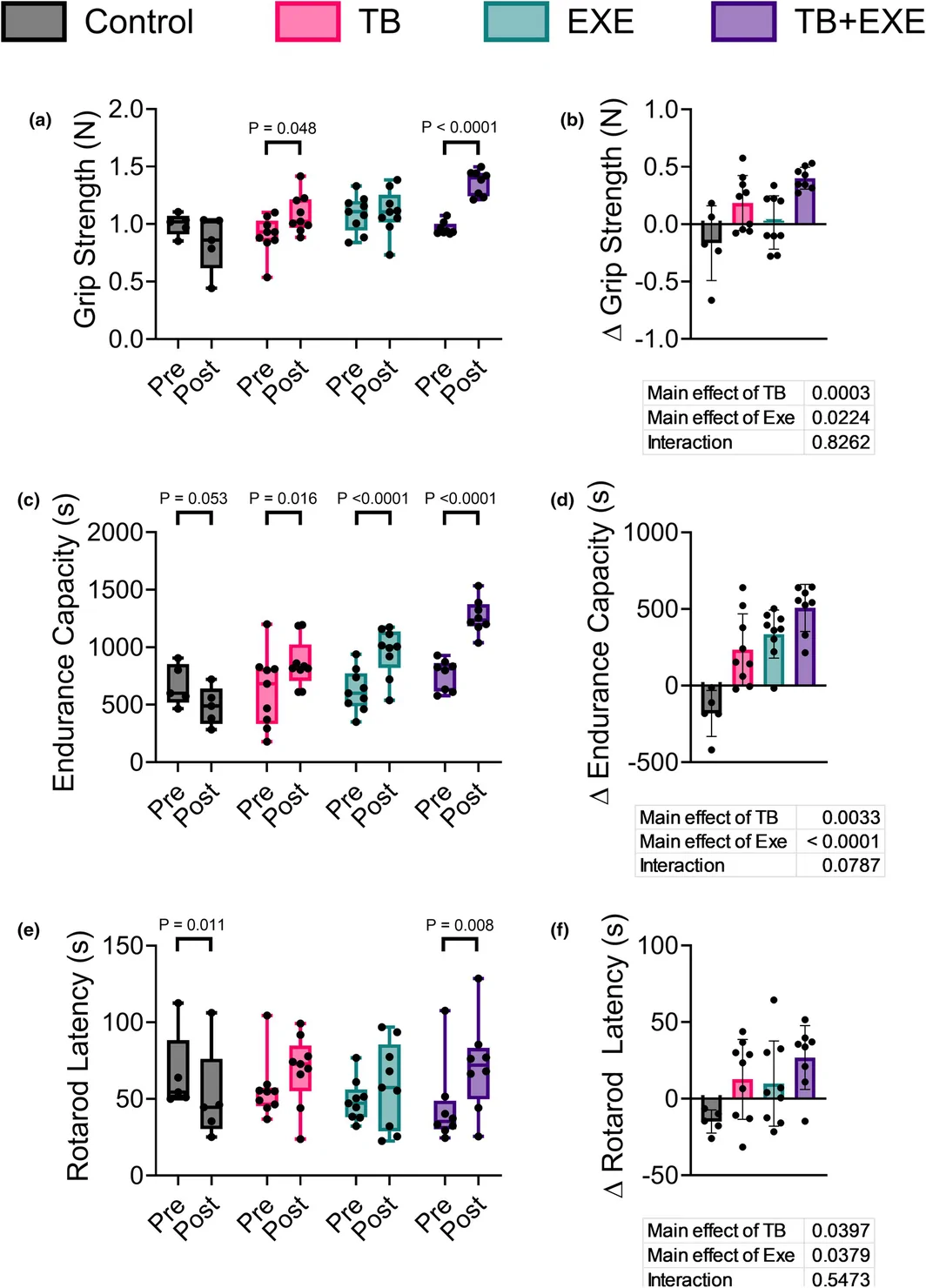
Tat‐Beclin1 enhances muscle strength and endurance and induces additive effects when combined with endurance training in aged mice. (a) Four‐paw grip strength (N) was measured before and after the intervention in Control, Tat‐Beclin1 (TB), Exercise (Exe), and TB+Exe groups. (b) Change in grip strength (Δ grip strength, N). (c) Endurance capacity, reported as time to exhaustion (s), was measured before and after the intervention. (d) Change in endurance capacity (Δ endurance capacity, s). (e) Rotarod performance, reported as latency to fall (s), was measured before and after the intervention. (f) Change in rotarod performance (Δ rotarod latency, s). Data are presented as mean ± standard deviation with individual data points shown. Pre‐ and post‐intervention measures were analyzed using a paired sample *t*‐test; data were considered statistically significant with an α < 0.05. Δ grip strength, Δ endurance capacity, and Δ rotarod latency were analyzed using two‐way ANOVA; data were considered significant for main effects or interaction with an α < 0.05. A Tukey's multiple comparison analysis was conducted when interaction was statistically significant. 23‐ to 24‐month‐old males C57BL6/J mice: Control, *N* = 5; TB, *N* = 9; Exe, *N* = 9; TB+Exe, *N* = 8.

### 3.3 Impact of Tat‐Beclin1, exercise, and their combination on the gastrocnemius/plantaris and tibialis anterior myofiber profile

To determine whether the short‐term TB+Exe alters myofiber size and type in the G/P and TA muscles, we examined muscle sections processed for myosin heavy chain (MyHC) types 2a, 2b, and 2x, and laminin. There were no differences in main effects or interaction in the G/P mean of myofiber cross‐sectional area (CSA–μm^2^) (Figure 4a,b, *p* > 0.05). There was a main effect of TB for G/P in the CSA of MyHC 2a (*p* = 0.0039) and 2X (*p* = 0.0336) (Figure 4a,c), and no statistical difference was observed in the G/P myofiber distribution (%) (Figure 4a,d, *p* > 0.05) among the groups. In the TA muscle, no statistical differences were observed in the mean myofiber CSA (μm^2^), CSA of each myofiber type, and myofiber distribution (Figure 4e–h, *p* > 0.05).

**FIGURE 4:**
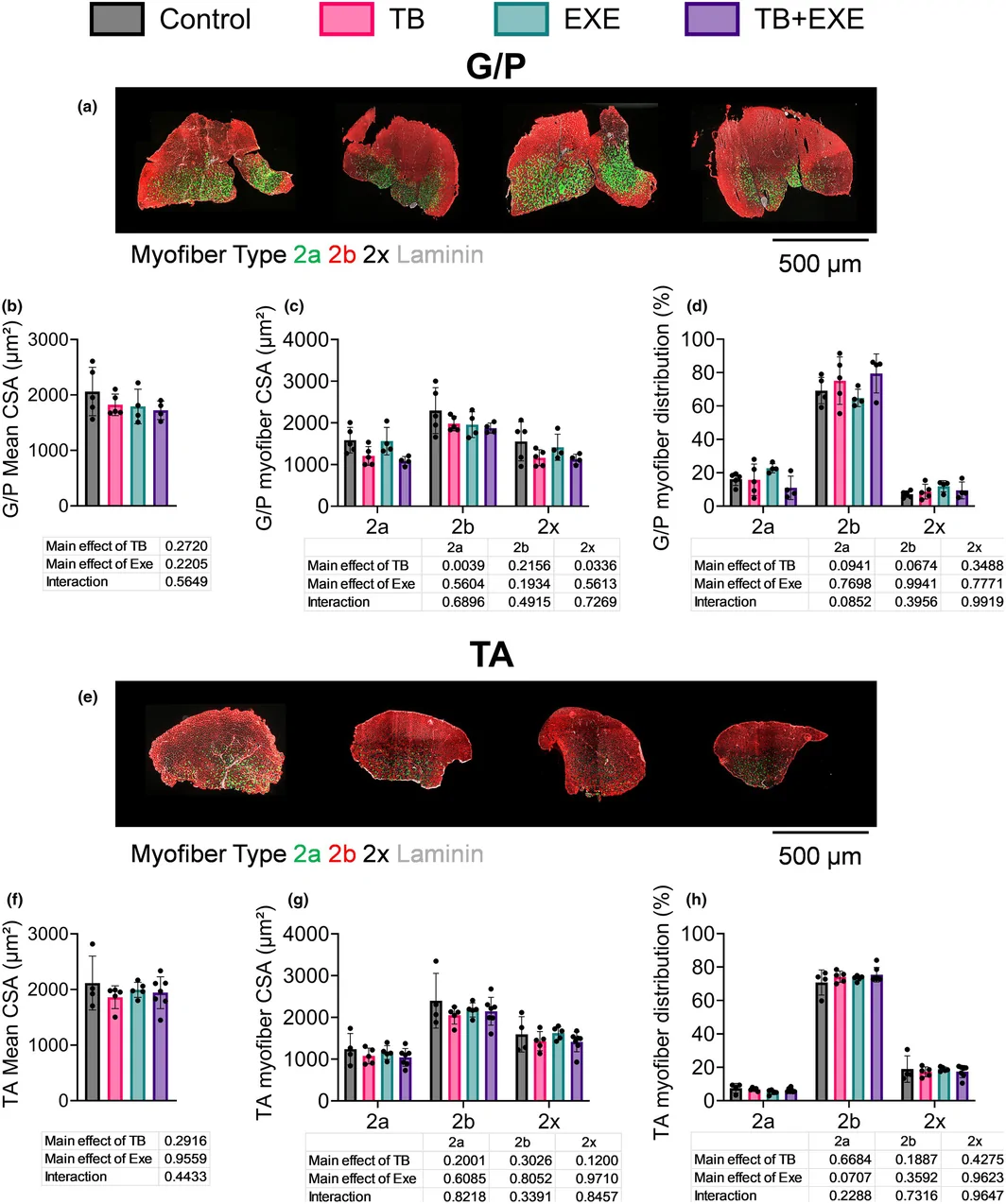
Gastrocnemius/plantaris and tibialis anterior fiber‐type composition and cross‐sectional area following Tat‐Beclin1 and endurance training. (a) Representative gastrocnemius/plantaris cross‐sections stained for myosin heavy chain fiber types, with type 2a fibers shown in green, type 2b fibers in red, type 2x fibers unstained/black, and laminin in gray outlining myofiber borders; scale bar, 500 μm. (b) Mean gastrocnemius/plantaris myofiber cross‐sectional area (CSA, μm^2^). (c) Gastrocnemius/plantaris myofiber CSA by fiber type (2a, 2b, 2x). (d) Gastrocnemius/plantaris myofiber‐type distribution (%). (e) Representative tibialis anterior cross‐sections stained as described above; scale bar, 500 μm. (f) Mean tibialis anterior myofiber CSA (μm^2^). (g) Tibialis anterior myofiber CSA by fiber type (2a, 2b, 2x). (h) Tibialis anterior myofiber‐type distribution (%). (i) Tibialis anterior myofiber‐type distribution (%). Data are presented as mean ± standard deviation with individual data points shown. Statistical analysis was conducted using two‐way ANOVA; data were considered significant for main effects or interaction with an α < 0.05. A Tukey's multiple comparison analysis was conducted when interaction was statistically significant. 23‐ to 24‐month‐old males C57BL6/J mice: Control, *N* = 5; TB, *N* = 5; Exe, *N* = 4; TB+Exe, *N* = 4, for G/P muscle analysis, and Control, *N* = 4; TB, *N* = 5; Exe, *N* = 5; TB+Exe, *N* = 7, for TA muscle analysis.

### 3.4 Protein expression of autophagy markers remains unchanged in the gastrocnemius/plantaris and tibialis anterior muscles after TB, Exe, or TB+Exe interventions

Given that our therapeutic approach combined Tat‐Beclin1 and endurance training, two well‐known autophagic agonists (Lira et al., 2013; Shoji‐Kawata et al., 2013), we assessed autophagy markers by western blotting in the G/P and TA muscles. Surprisingly, we found limited changes in the protein expression of the autophagic markers assessed, including Beclin1, LC3B‐II/I ratio, and p62, in the G/P muscle (Figure 5a–d). In the TA muscle, there was a significant decrease in Beclin1 expression (Figure 5e,f, interaction *p* = 0.0273) in the TB+Exe group, while no significant differences were observed in the LC3B II/I ratio and p62 expression (Figure 5e,g,h, *p* > 0.05).

**FIGURE 5:**
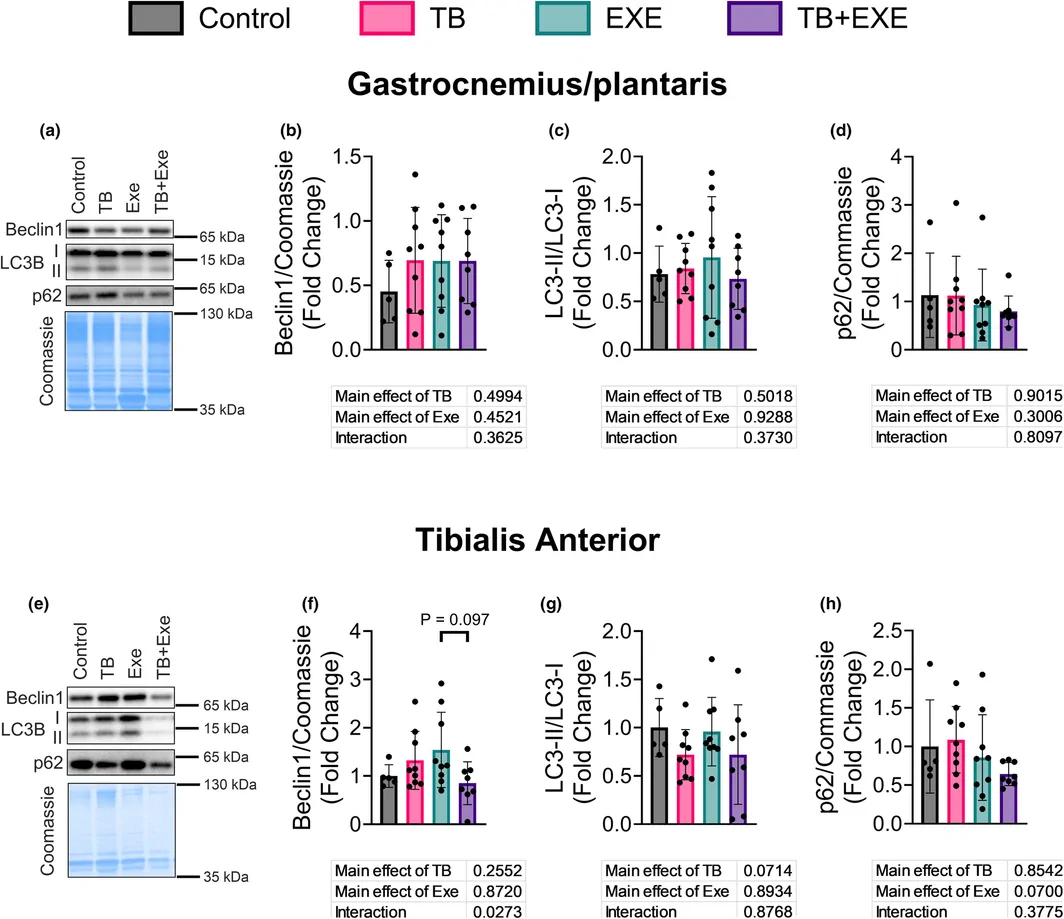
Autophagy‐marker protein abundance in gastrocnemius/plantaris and tibialis anterior after Tat‐Beclin1 and endurance training. Top panel, gastrocnemius/plantaris muscle: (a) Representative immunoblots for Beclin1, LC3B I and II, and p62, with Coomassie brilliant blue staining shown as a loading control. Quantification is shown for (b) Beclin1/Coomassie, (c) LC3‐II/LC3‐I ratio, and (d) p62/Coomassie. Bottom panel, tibialis anterior muscle: (e) Representative immunoblots for Beclin1, LC3B I and II, and p62, with Coomassie brilliant blue staining shown as a loading control. Quantification is shown for (f) Beclin1/Coomassie, (g) LC3‐II/LC3‐I ratio, and (h) p62/Coomassie. Data are presented as mean ± standard deviation with individual data points shown. Statistical analysis was conducted using two‐way ANOVA; data were considered significant for main effects or interaction with an α < 0.05. A Tukey's multiple comparison analysis was conducted when interaction was statistically significant. 23‐ to 24‐month‐old males. Control, *N* = 5; TB, *N* = 9; Exe, *N* = 9; TB + Exe, *N* = 8.

### 3.5 An overall upregulation in biological processes regulating fatty acid metabolism is commonly observed in TB, Exe, and TB+Exe groups

To investigate the mechanisms underlying functional adaptations induced by TB and Exe therapies, we performed a proteomic analysis of G/P muscles. Compared to the control group, the Gene Ontology biological processes (GO: BP) observed in each treated group present similarities (Figure 6a,c,e). For instance, there was an overall upregulation of fatty acid (FA) metabolism biological processes in TB (FA catabolic process, NES: 1.69, FDR < 0.10), Exe (Regulation of FA catabolic process, NES: 1.77, FDR < 0.10), and TB+Exe (FA β‐oxidation, NES: 1.90, FDR < 0.10). Considering the overall similarity of FA metabolism and the improved endurance capacity, a further comparison of differentially expressed proteins in this GO: BP relative to the control group revealed substantial overlap between TB and TB+Exe groups (14/20 proteins; 70% of the TB signature), whereas Exe shared only 2/20 proteins (10%) with TB and 3/25 proteins (12%) with TB+Exe. ACACB was the sole protein commonly upregulated in all three comparisons. ACACB (ACC2) is known to regulate FA entry into mitochondria (Abu‐Elheiga et al., 2000).

**FIGURE 6:**
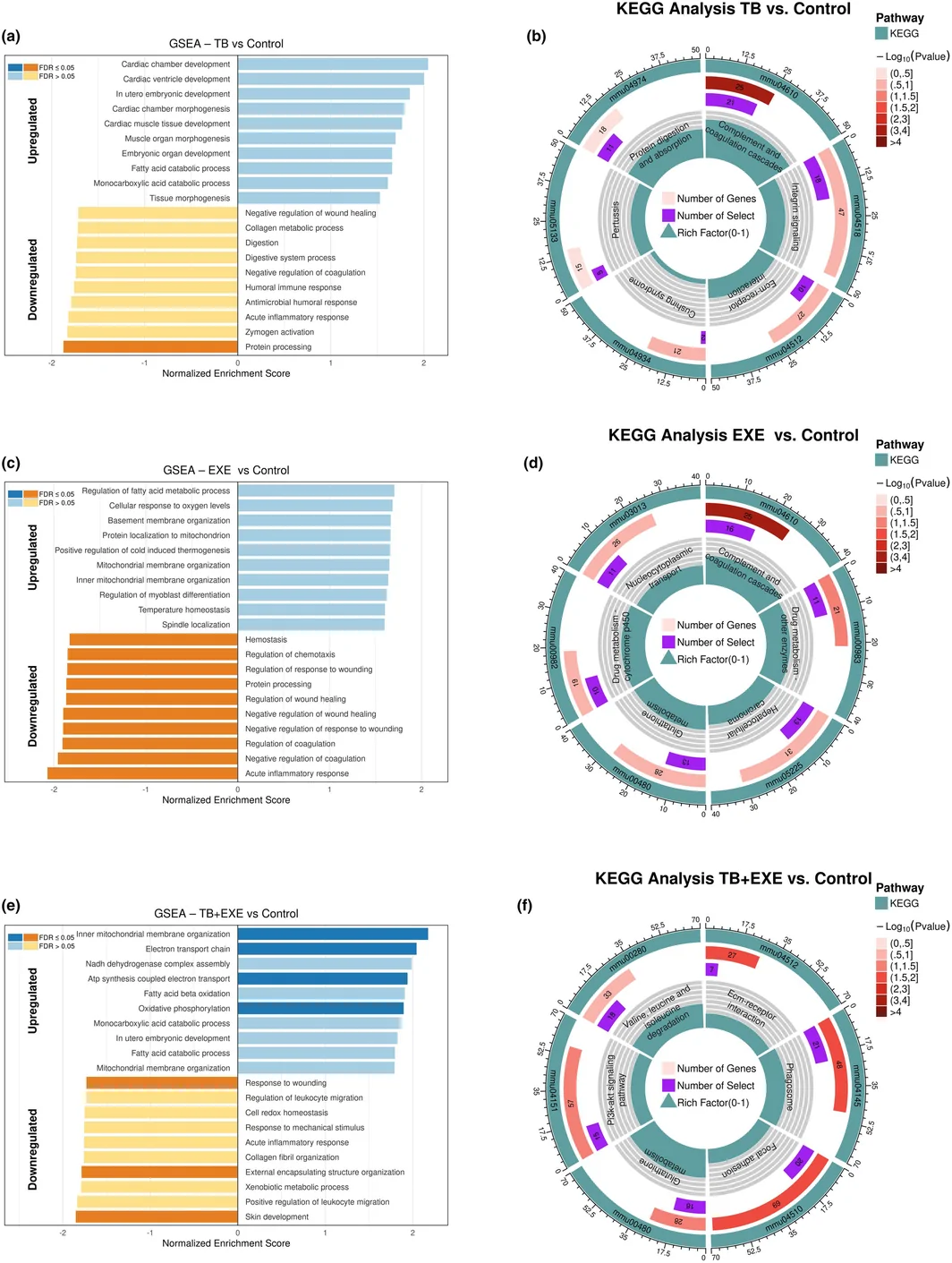
Gene Ontology Biological Process terms and KEGG pathway analysis for each treatment compared with Control. (a, c, and e) GSEA GO Biological Process terms showing the top upregulated and downregulated processes, ranked by Normalized Enrichment Score (NES), in TB versus Control, Exe versus Control, and TB+Exe versus Control, respectively. (b, d, and f) KEGG pathway analyses showing the top regulated pathways in TB versus Control, Exe versus Control, and TB+Exe versus Control, respectively. Analyses were corrected using the Benjamini–Hochberg false discovery rate (FDR < 0.1). 23‐ to 24‐month‐old male C57BL6/J mice used for proteomics: Control, *N* = 3; TB, *N* = 4; Exe, *N* = 4; TB + Exe, *N* = 4.

### 3.6 Proteomic signature of TB+Exe reveals upregulation of mitochondrial bioenergetic biological processes and an unexpected upregulation of acute inflammatory responses

The heatmap comparing the TB versus Exe groups showed a distinct protein pattern (Figure 7a). The GO: BP annotation showed upregulation of several muscle biology processes in the TB group versus the Exe group, including proteins involved in muscle contraction (NES: 1.84, FDR < 0.10) and adaptation (NES: 1.72, FDR < 0.10). For instance, TB increased the levels of troponin proteins, including TNNC1, TNNI2, and SCN4A, indicating changes in the regulation of muscle contraction (Figure 7b). The KEGG pathway analysis showed upregulation of proteins involved in energy pathways, including fatty acid and glutathione metabolism, as well as axon guidance (Figure 7c). When we compared TB+Exe with TB (Figure 7d–f), 1/3 of the proteins were upregulated in the TB+Exe group, as shown in the heatmap in Figure 7d. The annotated GO: BP revealed that the top 10 upregulated processes were related to mitochondrial bioenergetics, including OXPHOS (NES: 2.10, FDR ≤ 0.05), ATP synthesis coupled to the electron transport chain (NES: 2.07, FDR ≤ 0.05), and cellular respiration (NES: 2.0, FDR ≤ 0.05), suggesting high aerobic energy production to support muscle function in the TB+Exe group. Interestingly, while OXPHOS and axon guidance were highlighted in the KEGG analysis as well, KEGG identified an increase in the complement pathway resulting from the TB+Exe strategy versus TB (Figure 7f).

**FIGURE 7:**
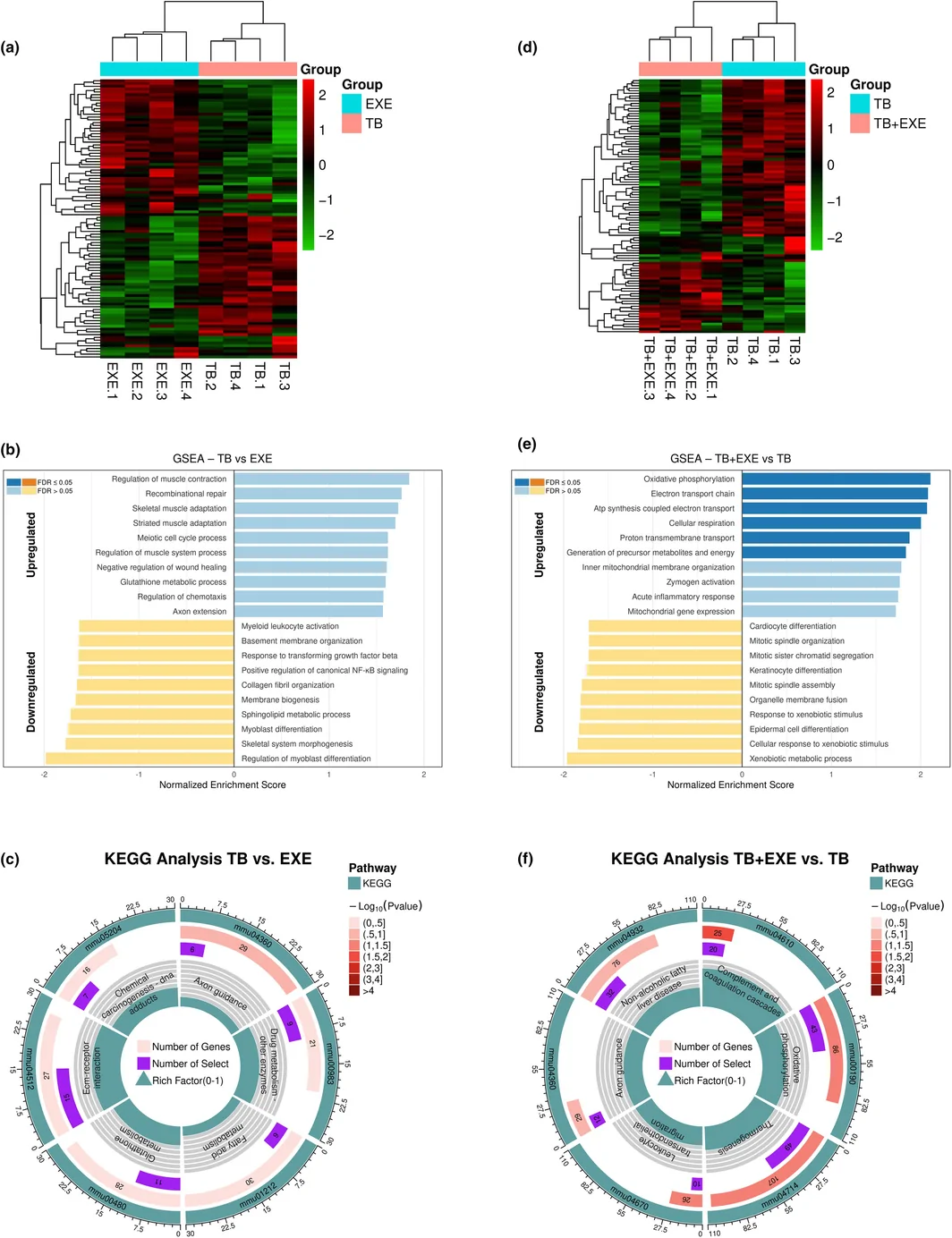
Proteomic signature of gastrocnemius/plantaris in TB versus Exe and TB+Exe versus TB. (a) Heatmap of the top 100 differentially abundant proteins comparing TB and Exe groups. (b) GSEA GO Biological Process terms showing the top upregulated and downregulated processes, ranked by Normalized Enrichment Score (NES), in TB versus Exe. (c) KEGG pathway analysis showing the top regulated pathways in TB versus Exe. (d) Heatmap of the top 100 differentially abundant proteins comparing TB+Exe and TB groups. (e) GSEA GO Biological Process terms showing the top upregulated and downregulated processes, ranked by Normalized Enrichment Score (NES), in TB+Exe versus TB. (f) KEGG pathway analysis showing the top regulated pathways in TB+Exe versus TB. Analyses were corrected using the Benjamini‐Hochberg false discovery rate (FDR < 0.1). 23‐ to 24‐month‐old males C57BL6/J mice used for proteomics: Control, *N* = 3; TB, *N* = 4; Exe, *N* = 4; TB+Exe, *N* = 4.

Moreover, the addition of Tat‐Beclin1 to endurance training revealed that approximately 1/3 of the proteins were upregulated compared to the Exe group (Figure 8a). While the annotated functional enrichment in the GO: BP analysis for the TB+Exe group versus the Exe group indicated upregulation of proteins involved in OXPHOS (NES: 1.65, FDR < 0.10) and downregulation of TGF‐β response (NES: −1.74, FDR < 0.10), it unexpectedly showed upregulation of proteins involved in stress and acute inflammatory responses (Figure 8b, NES: 1.83, FDR < 0.10). The KEGG pathway analysis revealed highly significant pathways involved in the organismal system (complement and coagulation) and the translational system (ribosome) (Figure 8c). Next, to identify the most statistically and biologically significant proteins in the GO: BP and KEGG analysis, we used a volcano plot (Figure 8d, *p* < 0.05 and Log_2_FC ≤ −1 or ≥ 1). Of the 2154 proteins detected in the G/P muscle, only a small number met the statistical threshold. Peripherin (PRPH), which is expressed in the neuromuscular junction (Manco et al., 2025), and mitochondrial ribosomal protein S7 (MRPS7), which is involved in protein synthesis (Hilander et al., 2024), were both downregulated (Figure 8d). Conversely, three proteins were upregulated in the TB+Exe group. We identified haptoglobin (HP), which has been shown to be required to prevent muscle atrophy (Bertaggia et al., 2014), inter‐alpha‐trypsin inhibitor heavy chain H4 (ITIH4), which has a general function in tissue repair (Wu et al., 2023), and orosomucoid‐1 (ORM1), a protein shown to improve endurance capacity in young rats and mice (Lei et al., 2016; Qin et al., 2016) (Figure 8d). This was further confirmed by WB analysis for HP and ORM1 in the G/P muscle of all four groups (Figure 8e). These proteins were chosen for their reported role in skeletal muscle atrophy and endurance (Bertaggia et al., 2014; Qin et al., 2016). Our data show a significant interaction (*p* = 0.0061) for Hp expression and revealed an increase in TB+Exe compared to Exe (Figure 8e,f; *p* = 0.0434). ORM1 protein expression showed a main effect of TB (Figure 8e,g, *p* = 0.0001).

**FIGURE 8:**
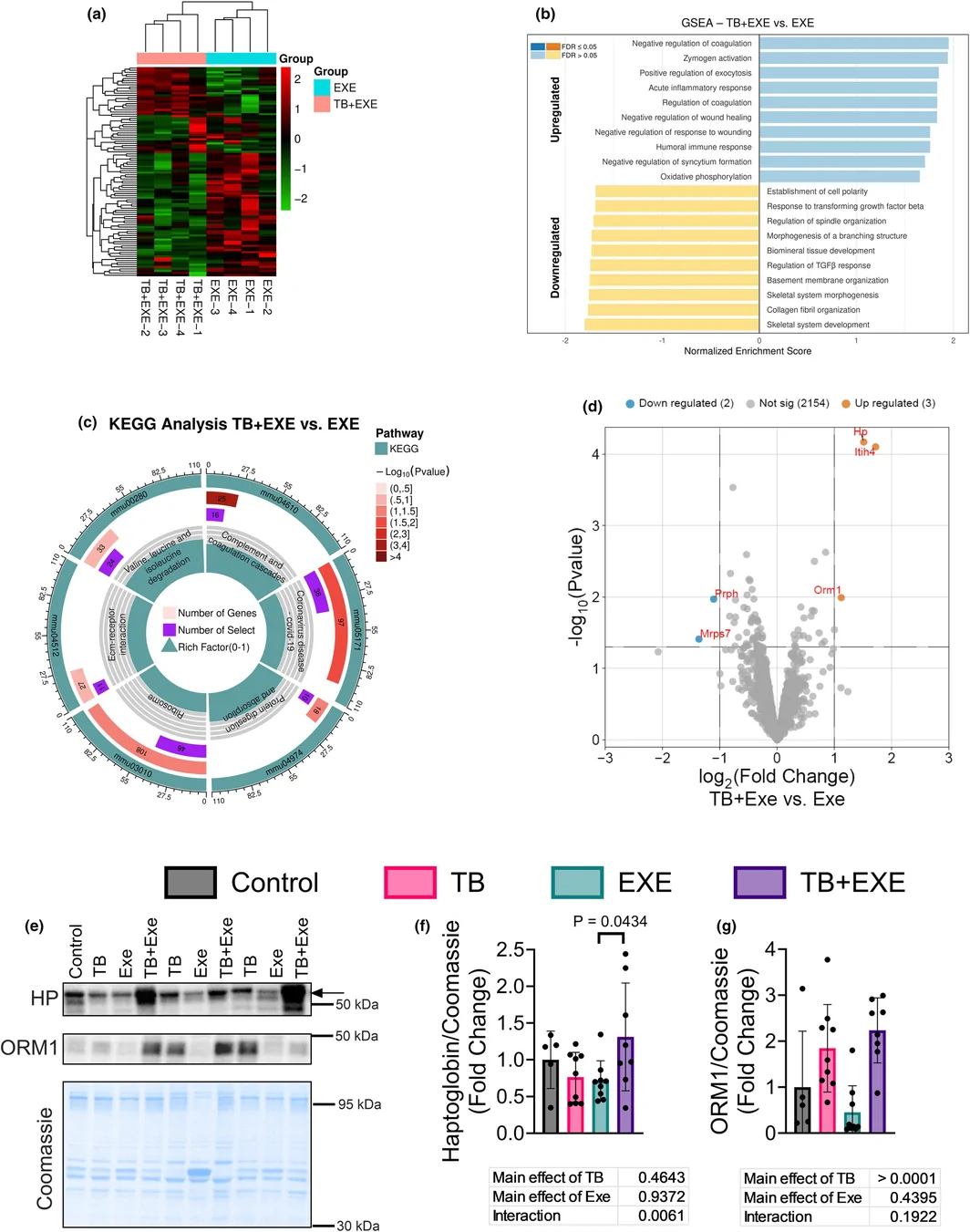
An acute‐phase/inflammatory molecular signature distinguishes TB+Exe from Exe and validates induction of haptoglobin and ORM1. (a) Heatmap of the top 100 differentially abundant proteins comparing TB+Exe and Exe groups. (b) GSEA GO Biological Process terms showing the top upregulated and downregulated processes, ranked by Normalized Enrichment Score (NES), in TB+Exe versus Exe. (c) KEGG pathway analysis showing the top regulated pathways in TB+Exe versus Exe. (d) Volcano plot of TB+Exe versus Exe showing log2 fold change versus −log10(*p* value), with significantly downregulated proteins shown in blue, significantly upregulated proteins shown in orange, and nonsignificant proteins shown in gray. 23‐ to 24‐month‐old males C57BL6/J mice. Proteomics sample sizes: Exe, *N* = 4; TB+Exe, *N* = 4. (e) Representative immunoblots for haptoglobin (HP) and orosomucoid‐1 (ORM1), with Coomassie brilliant blue staining as the loading control, across Control, TB, Exe, and TB+Exe groups. Quantification is shown for (f) HP/Coomassie and (g) ORM1/Coomassie. Data are presented as mean ± standard deviation with individual data points shown. Statistical analysis was conducted using two‐way ANOVA; data were considered significant for main effects or interaction with an α < 0.05. A Tukey's multiple comparison analysis was conducted when interaction was statistically significant. 23‐ to 24‐month‐old males C57BL6/J mice. Immunoblot sample sizes: Control, *N* = 5; TB, *N* = 9; Exe, *N* = 9; TB+Exe, *N* = 8.

## 4 DISCUSSION

To the best of our knowledge, the current study is the first to investigate the impact of Tat‐Beclin1 (TB), alone or in combination with endurance training, on the physical function of aged male mice. In this study, although no significant interaction was detected between TB and Exe, both factors independently improved grip strength and endurance capacity, as well as balance and coordination. The TB+Exe group showed further improvements that appeared to combine the effects of both interventions, suggesting an additive benefit. The lack of statistical interaction indicates that TB did not modify the effect of Exe beyond their individual contributions, yet the combined approach may offer incremental gains. In addition, proteomic profiling of the G/P muscle revealed that TB modulated proteins involved in muscle contraction, and that TB+Exe upregulated proteins associated with the acute inflammatory response. Our data suggest that this novel combinatorial strategy benefits muscle against age‐induced physical dysfunction, in part through an underexplored mechanism.

With advancing age, key physiological systems, including the cardiovascular, musculoskeletal, and nervous systems, begin to underperform, compromising the overall quality of life. In many preclinical studies, assessments of grip strength, time to exhaustion on a treadmill, and balance/coordination on a rotarod are used as affordable, rapid, and reliable methods to examine physical function (Bonetto et al., 2015; Clayton et al., 2022; Justice et al., 2014; Justice et al., 2016; Kerr et al., 2024; Murray et al., 2026). In this study, 23‐month‐old male mice from the TB and Exe groups independently increased four‐paw grip strength, endurance capacity, and balance and coordination. Interestingly, we observed considerable variability in grip strength, endurance, and rotarod performance, which likely reflects biological differences in aging, even among animals of the same strain and age. Despite using standardized testing and controls, individual baseline differences and responses to interventions may have contributed to the dispersion. Previous studies have shown promising outcomes with several agents in attenuating age‐related declines in physical function (Kerr et al., 2025; Long et al., 2017; Rolland et al., 2023; Xue et al., 2016). Among them, metformin, a widely used medication originally approved for treating type 2 diabetes, prevented muscle atrophy in older adults without diabetes after bed rest (Petrocelli et al., 2023). Rapamycin, a potent mammalian Target of Rapamycin Complex 1 (mTORC1) inhibitor, improves grip strength, total running time, and neuromuscular junction function in aged mice (Ham et al., 2022). Additionally, an umbrella review of several pharmacological interventions concluded that only vitamin D (VitD) for older women with severely low VitD levels and testosterone for older men with combined muscle weakness and low levels of testosterone are clinically justifiable for attenuating age‐related loss of muscle mass and function (i.e., sarcopenia) (De Spiegeleer et al., 2018). Although the focus of this study is on autophagy and skeletal muscle, we cannot rule out the contribution of different tissues to the observed physical function benefits. In fact, during exercise or Tat‐Beclin1 treatment, autophagy is regulated in several tissues and may contribute to cardiovascular, cognitive, and metabolic health (Kuramoto et al., 2023). Thus, the finding that TB improved domains of physical function directly affected by aging indicates that a pharmacological approach targeting Beclin1 is a feasible strategy to attenuate age‐related physical dysfunction.

While targeting aging‐related physical dysfunction with a standalone pharmacological approach is promising, replicating these benefits when combining these agents with regular exercise is challenging (Elliehausen et al., 2023). Metformin and rapamycin have been extensively studied in combination with regular exercise. When combined with progressive resistance exercise, metformin has been shown to antagonize the benefits of exercise in humans (Walton et al., 2019). In addition, metformin adversely affected aerobic exercise adaptation, primarily by attenuating muscle mitochondrial respiration in aged rodents and older adults (Bruss et al., 2025; Konopka et al., 2019). Similarly, rapamycin opposes muscle hypertrophy adaptations to chronic overload and resistance exercise (Bodine et al., 2001; Drummond et al., 2009). Intriguingly, the frequency of rapamycin administration seems to matter. The results presented in this study indicate that combining TB+Exe does not synergistically increase the assessed physical function outcomes, at least during a short‐term strategy, but notably, the addition of TB does not blunt endurance exercise adaptation as demonstrated in studies using metformin and rapamycin (Bodine et al., 2001; Bruss et al., 2025; Drummond et al., 2009; Elliehausen et al., 2023; Konopka et al., 2019; Walton et al., 2019). Moreover, a recent study (Elliehausen, Olszewski, Minton, Shult, et al., 2025) proposed two dosing schedules: 3x per week (frequent strategy) and 1× per week (intermittent strategy), both administered for 8 weeks in combination with progressive weighted wheel running (PoWeR), a high‐volume exercise modality shown to increase muscle mass and function (Elliehausen, Olszewski, Minton, Spiegelhoff, et al., 2025). The study demonstrated that the intermittent strategy, combined with PoWeR, produces promising outcomes in physical performance and muscle adaptations compared with a frequent‐dosing strategy. Therefore, the present study and others suggest that careful planning of dosing strategies should be incorporated into experimental designs to maximize the drug‐boosted increase in physical function when combined with regular exercise.

Endurance training and Tat‐Beclin1 may independently activate autophagy (Rocchi & He, 2017a, 2017b), yet our data show no overall change in the expression of autophagy markers (i.e., Beclin1, LC3B, and p62). A number of factors could have contributed to these outcomes in this study. For instance, the absence of assessment of LC3B‐II turnover and p62 degradation by autophagic flux assays. Examination of autophagic flux can be achieved by adding a separate cohort of animals, reconducting the TB, Exe, and TB+Exe experiments, and ultimately treating them with lysosomal inhibitors (e.g., chloroquine) or agents that block autophagosome‐lysosome fusion (e.g., colchicine) prior to euthanasia (Ju et al., 2010). Another possible explanation is the regulation of food intake prior to euthanasia, since rich nutrient conditions may blunt autophagic activity (Mizushima et al., 2010). In this study, we did not control the animals' food intake, which may have masked alterations in the autophagy markers. Together, these factors suggest that the limited to no changes in autophagy markers likely reflect limitations in detecting dynamic autophagic activity at the selected endpoint, rather than a true lack of autophagy induction by endurance training or Tat‐Beclin1.

Proteomic profiling demonstrated that overall, FA metabolism biological processes were similarly upregulated in all three treatments compared to the control group. This outcome suggests the improvements observed in endurance capacity were, at least in part, induced by upregulation of proteins within FA metabolism biological processes. In fact, elevated ACACB (ACC2) expression, a protein commonly upregulated in all treatments, suggests remodeling of skeletal muscle lipid metabolism and an increased capacity for fatty acid flux regulation (Lundsgaard et al., 2018). Since exercise primarily modulates ACC2 through AMPK‐dependent phosphorylation, higher protein levels may coexist with enhanced fatty acid oxidation. Next, we compared TB with Exe and observed upregulation of several biological processes related to skeletal muscle contraction and organization, including upregulation of the troponin isoforms TNNC1 and TNNI2, suggesting coordinated regulation of calcium binding and myosin‐actin interactions. Also, the upregulation of proteins controlling cytoplasmic calcium during the contraction of slow‐twitch and fast‐twitch fibers, like Atp2a2 (SERCA2) and Atp2a1 (SERCA1), suggests highly orchestrated improvements in muscle function within MyHC type 1 and 2. However, these molecular changes were not accompanied by alterations in muscle morphology.

The proteomic analysis performed in the G/P muscles from the TB+Exe group revealed increases in proteins involved in biological processes associated with mitochondrial energetics and the acute inflammatory response, as corroborated by the few proteins detected in the volcano plot and confirmed by the WB analyses. HP and ORM1 are of particular interest in the context of skeletal muscle. Although they have several biological functions, ranging from drug binding and anti‐sepsis to inhibiting superoxide production (ORM1) and from preventing excessive hemolysis to promoting antioxidant effects (HP), both proteins have been previously linked to physical function and muscle atrophy. A 5‐month‐old Hp‐knockout mouse model exhibited muscle atrophy, decreased muscle strength following an exercise challenge, and impaired physical performance (Bertaggia et al., 2014), indicating the requirement for Hp to maintain physical function. ORM is emerging as an essential regulator of glucose and lipid metabolism (Zhang et al., 2026). Treating young rodents with ORM1 increased endurance capacity and muscle glycogen content (Lei et al., 2016). Additionally, in aged mice (28‐month‐old) subjected to 26 months of caloric restriction, known to activate autophagy, ORM1 was identified as a hub gene uniquely associated with caloric restriction (Altab et al., 2025). Mechanistically, it has been shown that ORM1 improves muscle endurance by activating AMPK, an energy sensor activating autophagy (Qin et al., 2016). However, the association between these proteins and autophagy remains elusive, at least in mammals, since ORM1 has been reported to be a selective autophagic receptor that interacts with ATG8 (LC3 ortholog) to activate autophagy in plants (Yang et al., 2019).

Collectively, our findings identify TB as a promising therapeutic agent to enhance physical function in aged male mice. They also indicate that adding TB to endurance training in an alternating‐day regimen does not blunt exercise adaptation and that although there was no synergistic outcome, TB+Exe showed great absolute values, which corroborates with an outcome comparable with additive effects of both therapies. Proteomic profiling indicates that all treatments led to an upregulation of FA metabolism, which is associated with improved physical function. Meanwhile, TB+Exe underscores the involvement of mitochondrial bioenergetic pathways and an unexpected acute inflammatory response signature (e.g., HP and ORM1), suggesting a potentially underexplored mechanism of muscle protection during age‐related decline in physical function.

### 4.1 Limitations of the present study and future directions

Our study provides new data that may pave the way for the inclusion of TB as a drug attenuating age‐related physical dysfunction, especially by not blocking exercise adaptations when combined with endurance training. Nevertheless, we acknowledge a few limitations. We found no changes in autophagy markers following TB or TB+Exe treatment. Future studies should consider food removal for at least 6 h prior to euthanasia. This would attenuate the confounding effects of recent food intake, since autophagy is finely regulated by nutrient status. Autophagy is a highly dynamic process, and adding a separate cohort treated with colchicine or chloroquine to assess autophagic flux should be considered in future studies. Moreover, given that age‐related physical function deterioration is a slow, progressive process, future studies should determine whether long‐term treatment with TB would elicit further improvements. In this study, we used only mice aged 23–24 months, roughly 69 years in human age, and future studies should include adult animals (4–6 months old) to further compare the impact of TB and TB+Exe on physical function. We recognize the lack of female animals in our study. This study, to the best of our knowledge, is the first to investigate the combination of Tat‐Beclin1 and endurance training in aged mice. Due to limited resources, male mice were studied first, but we are currently conducting similar studies in females. Finally, prospective studies focusing on detected acute inflammatory proteins, including orosomucoid‐1, are necessary to determine the benefits of TB+Exe and to assess whether it can serve as a standalone therapy for attenuating age‐related decline in physical function.

## AUTHOR CONTRIBUTIONS

**Thuan Thien Tchen:** Conceptualization; data curation; formal analysis. **Shakibur Rahman:** Data curation. **Thaysa Ghiarone:** Data curation; formal analysis. **Lynn A. Spruce:** Formal analysis. **Hossein Fazelinia:** Formal analysis. **Elizabeth M. Brown:** Formal analysis. **Charalampos Papachristou:** Data curation; formal analysis; methodology. **Sue C. Bodine:** Conceptualization. **Vitor A. Lira:** Conceptualization. **Kleiton A. S. Silva:** Conceptualization; data curation; formal analysis; funding acquisition; investigation; project administration; supervision.

## FUNDING INFORMATION

This work was supported by the Camden Health Research Initiative (CHRI) and the NIH NIGMS award R16GM159126 and NIH NIA award R15AG095821 to KASS.

## CONFLICT OF INTEREST STATEMENT

The authors declare no conflict of interest.

## ETHICS STATEMENT

This work was approved by the Rowan University IACUC under the protocol number: 2022‐1316. This work folloed the Principles for the utilization and care of vertebrate animals used in testing, research and training.

## Supporting information


Figure S1.

## Data Availability

The mass spectrometry proteomics data have been deposited to the ProteomeXchange Consortium via the PRIDE partner repository with the dataset identifier PXD083861.
