# Supplementary material for: Adding Tat‐Beclin1 to an endurance training program promotes additive improvements in physical function in aged male mice
Source: Physiol Rep. 2026 Oct 9;14(19):e71128. doi: 10.14814/phy2.71128 (PMC13652958; doi:10.14814/phy2.71128)
Supplement: Supplementary file 1 [file PHY2-14-e71128-s001.pdf]

Supplemental Figure S1A

Orosomucoid-1 (ORM1)
Western blotting from Liver (left panel) and from G/P muscle (right panel – From Figure 8E)

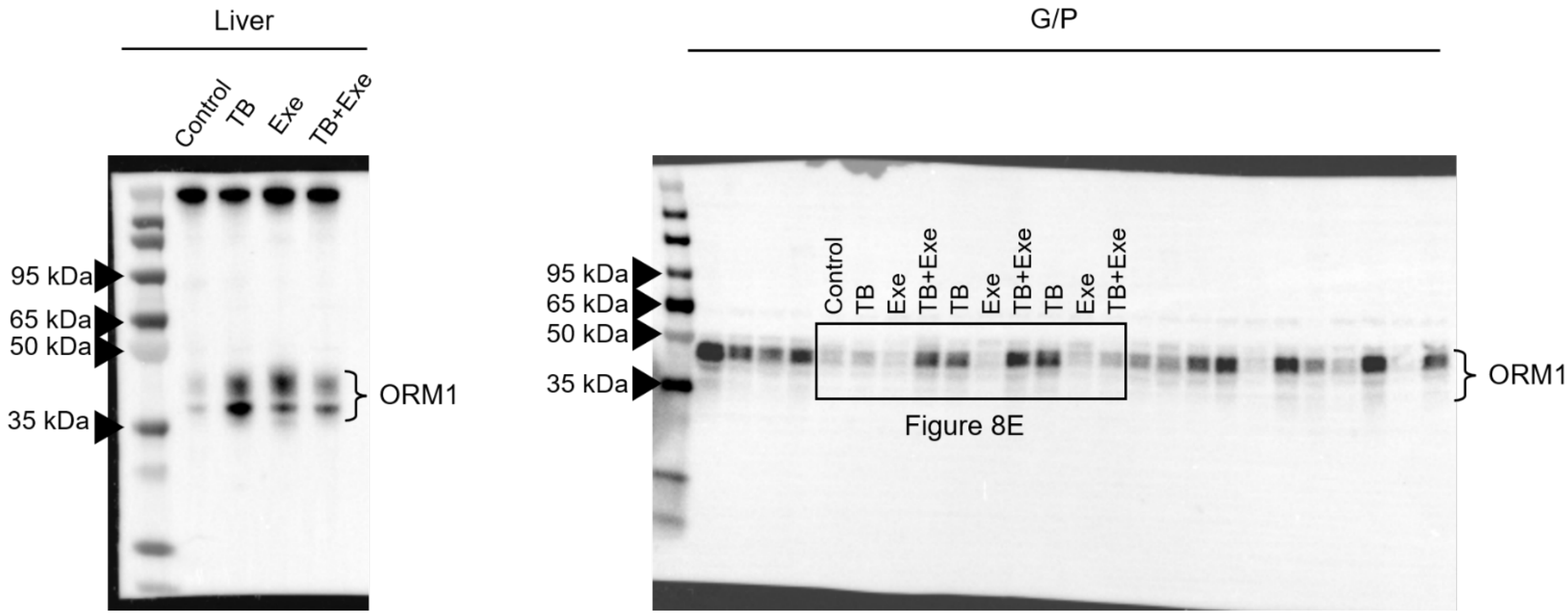

Supplemental Figure S1B

Haptoglobin (HP)
Western blotting from Liver (left panel) and from G/P muscle (right panel – From Figure 8E)

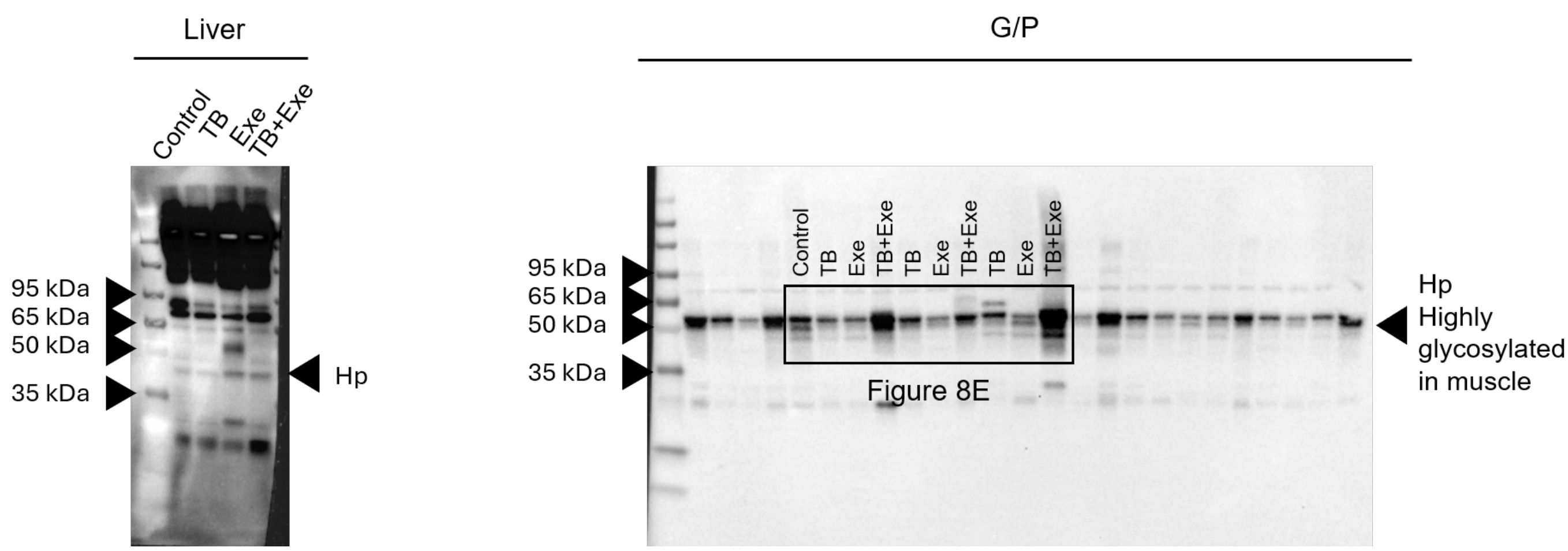

Supplemental Figure S1: (S1A) Representative image from Orosomucoid-1 western blotting in the liver (left panel) and gastrocnemius/plantaris muscle (right panel). (S1B) Representative image from Haptoglobin western blotting in the liver (left panel) and gastrocnemius/plantaris muscle (right panel). We used the bands indicated in the gastrocnemius/plantaris panels for orosomucoid-1 and haptoglobin in Figure 8E, shown in the main figures.
